# Supplementary material for: A comprehensive framework for the interpretation of TTN missense variants
Source: Genome Med. 2026 Feb 26;18:32. doi: 10.1186/s13073-026-01605-1 (PMC13007378; doi:10.1186/s13073-026-01605-1)
Supplement: Supplementary file 2 — Additional file 2. Supplementary clinical, histological, and functional data. [file 13073_2026_1605_MOESM2_ESM.docx]

**Additional File 2. Supplementary clinical, histological, and structural variant data**

**Index**

Fig S1. Muscle biopsy from Patient F1-P1.

Fig. S2. Muscle biopsy of Patient F7-P1.

Fig. S3. Muscle MRI of Patient F8-P1.

Fig. S4. Experimental or computationally predicted structures of titin domains (Ig-31, Ig-54, and Ig-68) studied in vitro

Fig. S5. Quotient surface accessible surface area q(SASA) of wild type (WT) amino acids mutated to either proline or any other amino acid in patient cohort

Fig. S6. Widefield fluorescence microscopy images of COS-7 cells expressing GFP-tagged WT and missense variant-containing titin domains.

Fig. S7. Proportions of predicted benign, indeterminate, and pathogenic *TTN* missense variants per exon based on AlphaMissense score.

Fig. S8. Full western blot showing assessment of solubility of WT and missense variant-containing titin domains.

**
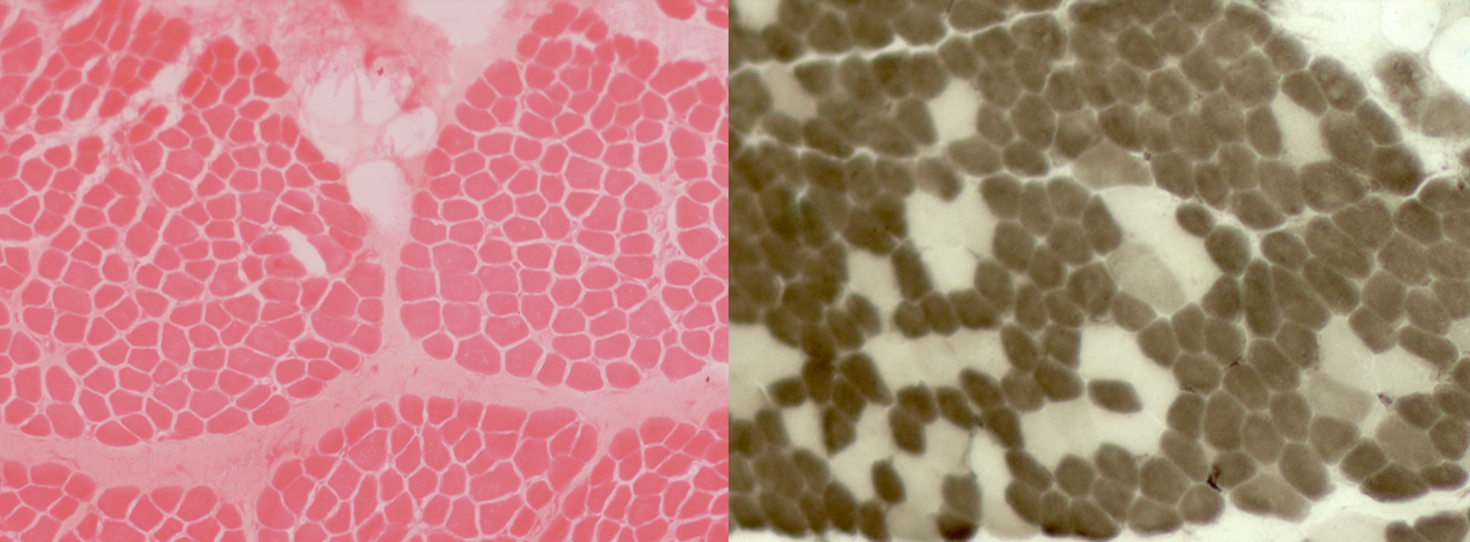
**

**Fig. S1.** Patient F1-P1, muscle biopsy. Vastus lateralis, performed in infancy.

Variability in muscle fibre calibre due to the presence of some atrophic fibres and a slight increase in endomysial connective tissue. ATPase pH 4.3 shows predominance of type 1 fibres.

**Fig. S2.** Patient F7-P1, showing distal involvement, scapular winging. Muscle biopsy (NADH staining) showing scattered atrophic fibers with structural disorganization and minicores (indicated by stars).


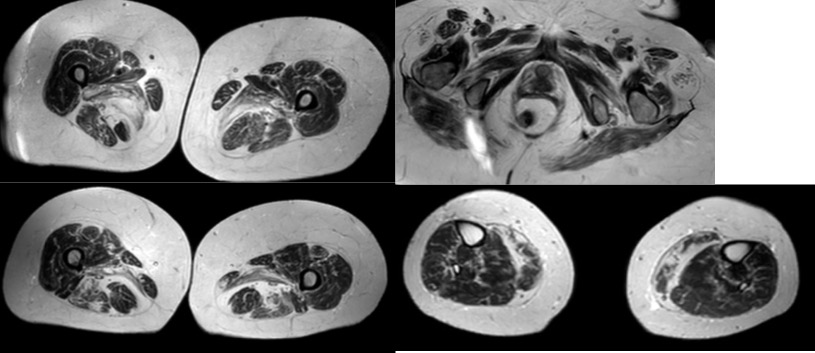


**Fig. S3.** Patient F8-P1, muscle MRI. Marked fatty replacement and atrophy of posterior thigh and calf muscles, with relative sparing of anterior compartments.

**
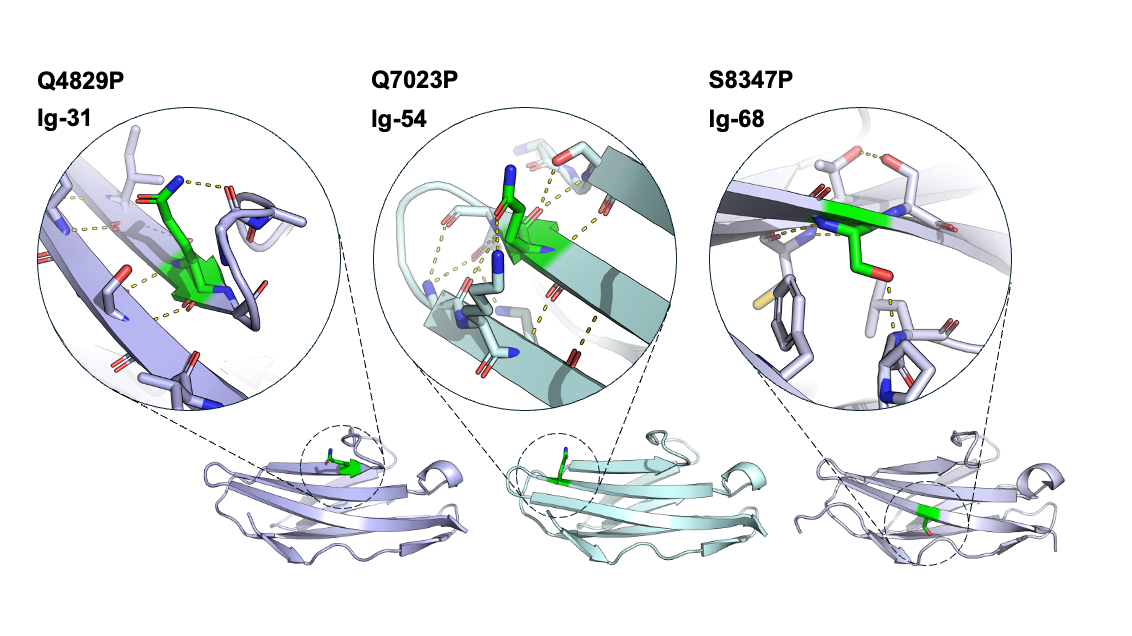
**

**Fig. S4**. Experimental or computationally-predicted structures of titin domains studied in vitro showing the atomic environment of residues mutated in patient missense variants to proline. The amino acids mutated in patients are shown as green sticks, with surrounding main-chain and side-chain atoms of interest also shown as sticks. Polar contacts between these residues are shown as dashed yellow lines. Ig-31 and Ig-54 are AlphaFold-predicted models downloaded from TITINdb. Ig-68 is from an experimental structure, with PDB code 3B43. Each of the three residues mutated in patients are at a different structural position, but all are located on a beta strand. Proline is typically incompatible with beta strands – it is a ”beta-breaker”.

**Fig. S5.** Quotient surface accessible surface area q(SASA) of amino acids mutated to either proline or any other amino acid in patient cohort, calculated from predicted or experimental domain structures. Amino acids with q(SASA) values above or below 0.3 are defined as surface or core residues, respectively. q(SASA) value retrieved from TITINdb (<https://titindb.kcl.ac.uk/TITINdb/> ).


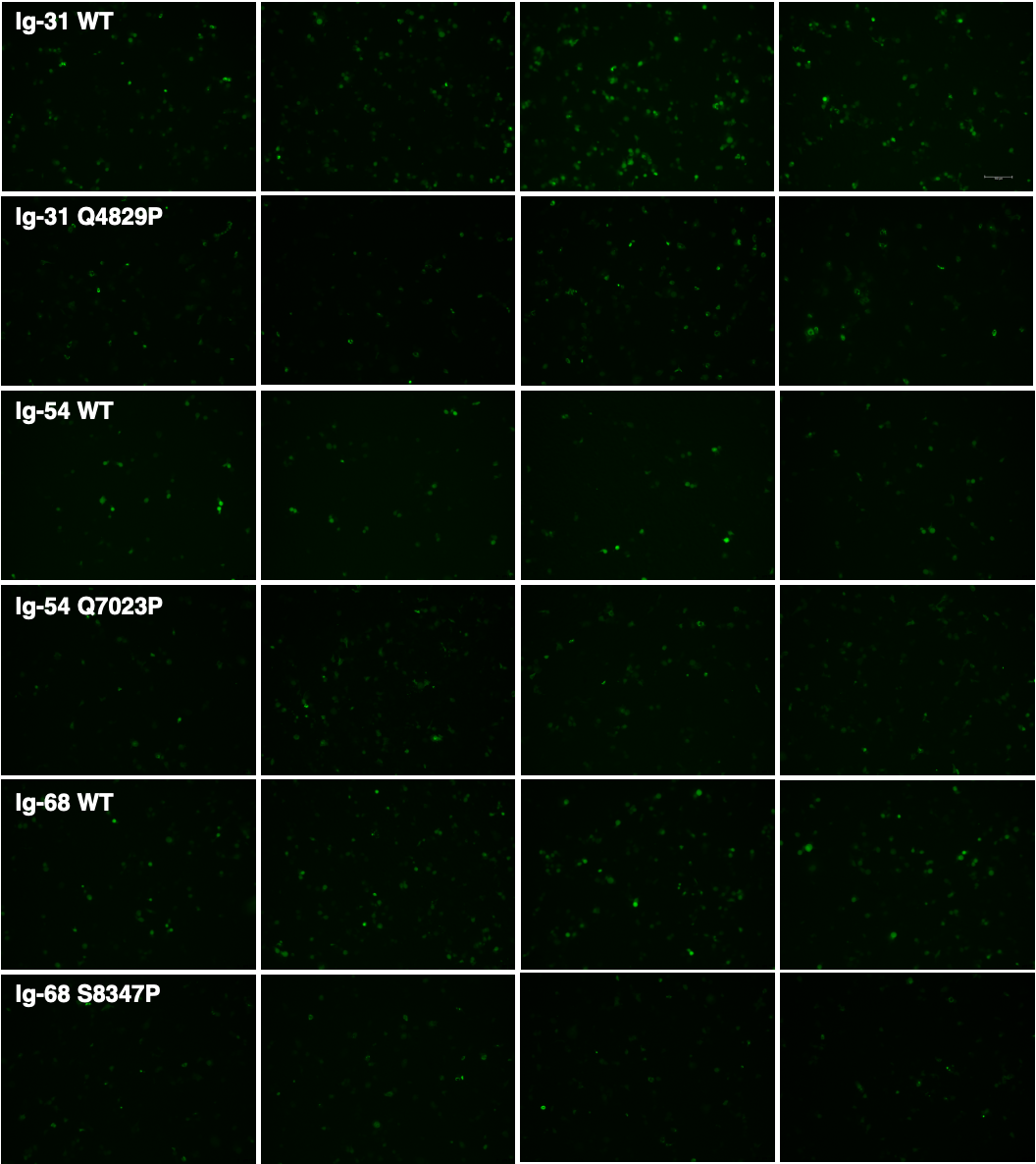


**Fig. S6.** Widefield fluorescence microscopy images of COS-7 cells expressing GFP-tagged WT and missense variant-containing titin domains. These images were used for the images analysis shown in Fig. 5B. Scale bar = 150um.


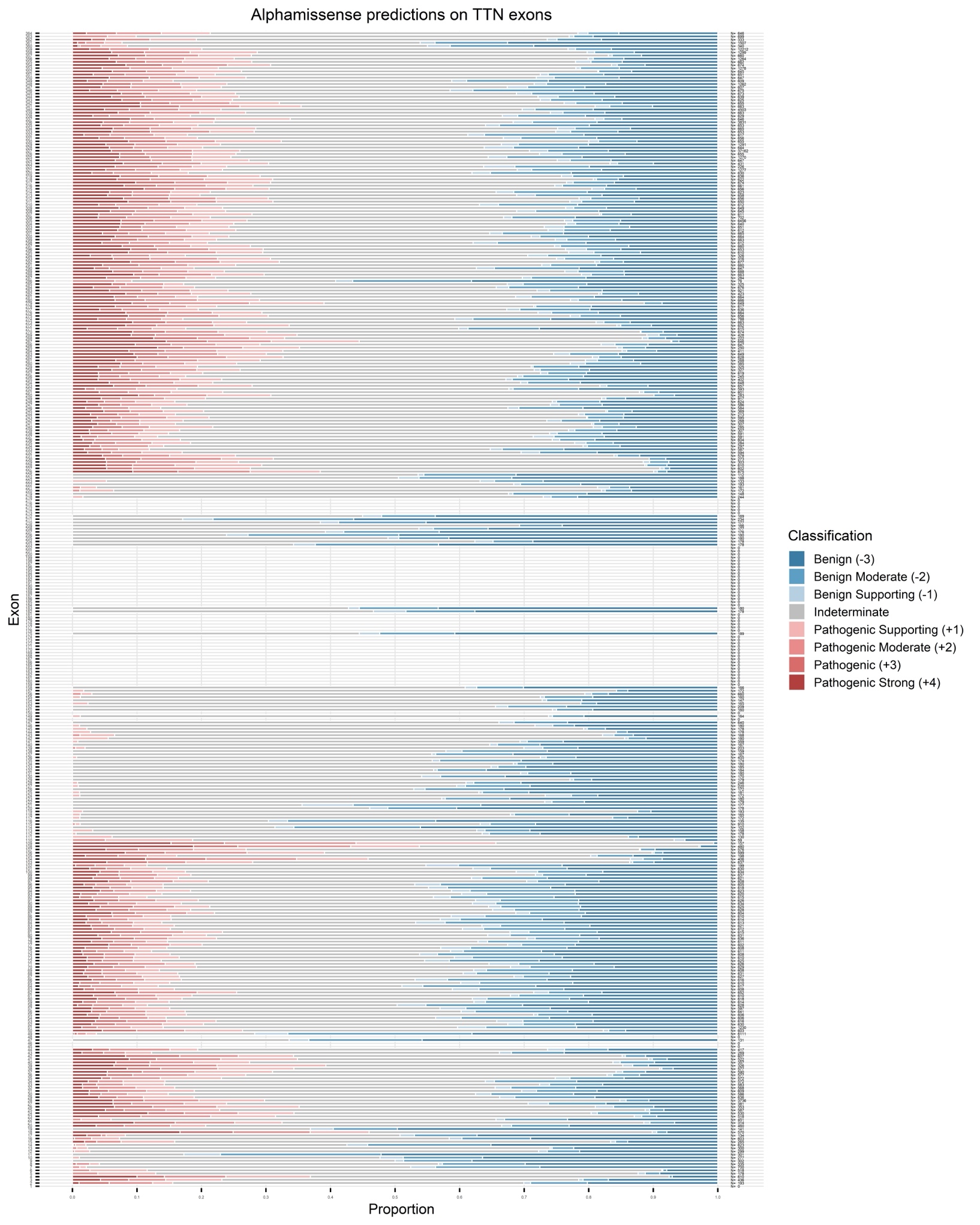


**Fig. S7.** Proportions of missense variants per *TTN* exon classified as benign (blue), indeterminate (grey), or pathogenic (red), according to AlphaMissense predictions. Categories follow ClinGen-recommended score thresholds described in the Methods section. Exons are shown from exon 1 at the bottom to exon 364 at the top.


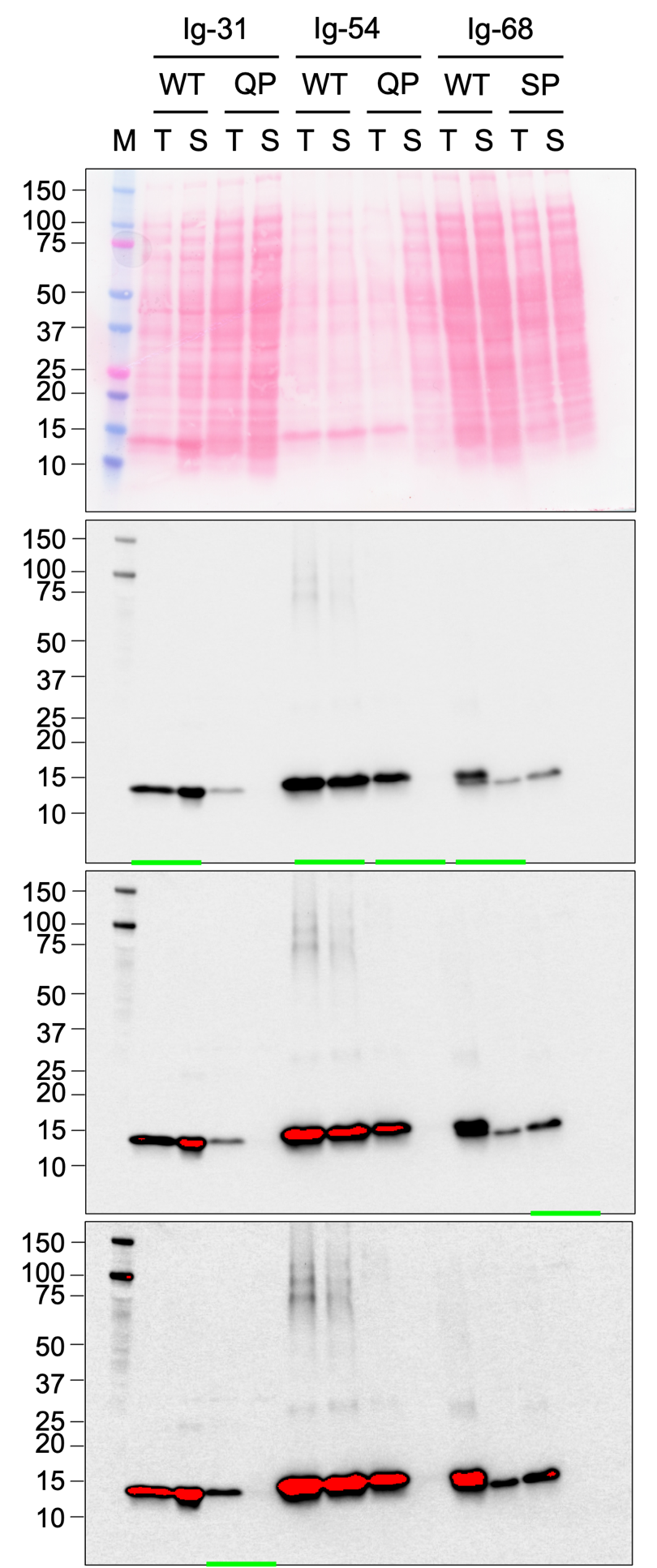


**Fig. S8.** Western blot probing presence of His-tagged titin domains Ig-31 WT and Q4829P, Ig-54 WT and Q7023P, and Ig-68 WT and S8347P in the soluble fraction of bacterial expression lysate. T = total lysate, S = soluble fraction, M = molecular weight marker. Exposure times used for each total lysate – soluble fraction pair are underlined in green.
