# Supplementary material for: A comprehensive framework for the interpretation of TTN missense variants
Source: Genome Med. 2026 Feb 26;18:32. doi: 10.1186/s13073-026-01605-1 (PMC13007378; doi:10.1186/s13073-026-01605-1)
Supplement: Supplementary file 5 — Additional file 5. ACMG/AMP evidence supporting the reclassification of p.(Gln7023Pro) and p.(Arg25480Pro) as likely pathogenic. [file 13073_2026_1605_MOESM5_ESM.docx]

| ACMG criterion | p.(Gln7023Pro) | p.(Arg25480Pro) | Evidence and rationale |
| --- | --- | --- | --- |
| PM3 (Detected in trans with pathogenic variant) | Moderate (1.25 points) | Supporting (0.50 points) | Evidence weighted following ClinGen recommendations for large genes (0.25 points per proband). Variant p.(Gln7023Pro) was observed in six unrelated affected families; p.(Arg25480Pro) in two unrelated families. |
| PS3 (Functional studies supportive of damaging effect) | Moderate | Moderate | Functional assays, previously used for other *TTN* variants with established pathogenicity, were performed. |
| PP1 (Co-segregation with disease in families) | Strong | Supporting | For p.(Gln7023Pro), co-segregation across 7 informative meioses in 5 families. (In Family 1, two affected siblings carry p.(Gln7023Pro), with confirmed parental segregation; in Family 2, an unaffected sibling carries only one variant, consistent with recessive inheritance.) For p.(Arg25480Pro), co-segregation across 2 informative meioses in 2 families. |
| PP3 (Computational evidence) | Supporting | Strong | AlphaMissense score = 0.83 for p.(Gln7023Pro) and 0.99 for p.(Arg25480Pro), exceeding ClinGen-recommended thresholds for PP3 activation. |
| PM2 (Absent or rare in population databases) | Supporting | Supporting | gnomAD genomes: homozygous allele count = 0 (<2) for TTN (AD/AR gene); overall high coverage (31.8×). Variant p.(Arg25480Pro) not detected in gnomAD genomes. |
| PP4 (Patient phenotype specific for disease) | Supporting | Supporting | Clinical features, muscle biopsy findings, and disease progression are consistent with recessive congenital titinopathy. |
| BP1 (Missense variant in a gene where LOF is known mechanism) | Deactivated | Deactivated | BP1 was manually removed: although TTN is historically associated with truncating variants, disease-causing missense variants have been reported (e.g., exons 344 and 364). Thus, the underlying assumption for BP1 does not hold. |
